# Higher rate of microscopic hematuria in elderly patients who take regular doses of aspirin: Result from AHAP Study

**Published:** 2016

**Authors:** Emadouddin Moudi, Seyed-Reza Hosseini, Ali Bijani

**Affiliations:** 1 Department of Urology, Shahid Beheshti Hospital, Babol University of Medical Sciences, Babol, Iran.; 2 Department of Community Medicine, Social Determinants of Health (SDH) Research Center, Babol University of Medical Sciences, Babol, Iran.; 3 Social Determinants of Health Research Center, Health Research Institute, Babol University of Medical Sciences, Babol, Iran.

**Keywords:** Aspirin, Elderly, Hematuria, Prevalence

## Abstract

**Background:**

Aspirin is the most widely used drug in medicine for cardiovascular and as recently for its role in cancer prevention. Although the risk of bleeding events increased following regular use of aspirin, little is known about the association of aspirin and hematuria. The present study aimed to evaluate the association of regular aspirin use and microscopic hematuria in elderly.

**Methods:**

In this study, we have extracted the data of elderly people who participated in Amirkola Health and Aging Project (AHAP) and taking regular doses of aspirin. The prevalence of microscopic hematuria was compared between the elderly who took aspirin regularly and those who did not take it.

**Results:**

A total of 1243 individuals (54.22% males, 45.78% females) were entered in to the study. Two hundred and eighty-four (23%) elderly took regular doses of aspirin. Microscopic hematuria was seen in 305 (24.54%) elderly. The prevalence of microscopic hematuria was 27.27% in regular users of aspirin and 23.72% in non-users of aspirin (P=0.126). The prevalence of microscopic hematuria was significantly higher among the regular users of aspirin compared to non-users in multiple logistic regression analysis (P=0.035, OR=1.40, 95%CI: 1.02-1.92).

**Conclusion:**

Taking regular doses of aspirin was accompanied with higher rate of microscopic hematuria in the elderly.

Regular use of aspirin is one of the most common preventive strategies against cardiovascular diseases (1-5). More recently; its preventive effect in various types of cancers has emerged and therefore has supported its routine use in high risk populations (6-8). It is estimated that more than 35% of adults in the United States take daily doses of aspirin (5, 9). Despite useful effects of aspirin, certain complications such as gastrointestinal bleeding, hemorrhagic stroke and bronchospasm are prevalent among the regular users of aspirin (10). Aspirin interferes with the aggregation of platelets via inhibiting cyclooxygenase-1 and 2 (COX-1 and 2) enzymes. Inhibition of COX-1 and 2 enzymes leads to decrease formation of thromboxane -A_2 _which plays a key role in platelet aggregation. Therefore, aspirin-users are at increased risk of bleeding events. However, the relationship between regular use of aspirin and hematuria is not well-studied yet. The present study aimed to compare the prevalence of microscopic hematuria in the elderly who took regular doses of aspirin and those who did not take it regularly. 

## Methods

**Study population and data collection:** We extracted data on the aspirin use and prevalence of microscopic hematuria among elderly individuals who were recruited in Amirkola Health and Ageing Project (AHAP) (11). The protocol of the AHAP study has been fully described elsewhere. Briefly, health status of 1616 individuals aged 60 years or more were screened and their data were collected prospectively within a two-year period (2011-2012). 

Several variables including demographic characteristics, body mass index (BMI), smoking status, chronic comorbidities (diabetes mellitus, hypertension, cardiovascular diseases etc.) and medicine use were evaluated and registered in AHAP database.

We have extracted the demographic data (age and gender), BMI, smoking history, history of diabetes, hematuria in urine analysis and the regular use of aspirin from the AHAP database using special checklists. The regular use of aspirin was defined as checking “yes” on the form of medication for daily use by the participant (self-reported). 

Microscopic hematuria was defined as the presence of 5 red blood cells or more per high-power fieled in urine microscopic analysis. We excluded the elderly with self-reported history of urologic disorders (gross hematuria, urolithiasis, cancers of urogenital system), kidney disorders, taking anticoagulation and other antiplatelet medication and individuals with incomplete registry data from the study. Finally we have calculated the prevalence of microscopic hematuria among aspirin users and non-users applying multivariate statistical model. 

**Statistical Analysis:** Data were analyzed using SPSS software Version 17 (IL, USA). T-test, chi-square, Fisher’s exact test were applied for comparing the frequency of microscopic hematuria among users and non-users of aspirin regarding their demographic characteristics, diabetes and smoking status. Multiple logistic regression analysis was used to adjust for potential confounding variables to isolate the association of aspirin use and the prevalence of microscopic hematuria. The p-value<0.05 was considered significant.

## Results

Among the 1616 individuals who participated in AHAP, we excluded 373 persons who met the exclusion criteria. A total of 1243 elderly entered the final analysis of whom 674 (54.22%) were males and 569 (45.78%) were females. Two hundred and eighty four (23%) individuals reported regular use of aspirin and 957 (77%) did not report regular use of aspirin. 

Demographic characteristics of the study population were shown in table 1.

**Table 1:** Demographic characteristics of elderly with and without regular use of aspirin (n=1243)

**Variables**	**Regular use of aspirin**		**P-value**
	**Yes (n=286)**	**No (957)**	
**Gender**			
Male (%) Female (%)	147 (21.8) 139 (24.4)	527 (78.2) 430 (75.6)	0.274
Age (mean±SD)	69.24±7.04	69.24±7.55	0.991
BMI (mean±SD)	28.11±4.18	26.75±4.63	<0.001
**History of diabetes**			
Yes (%) No (%)	119 (30.9) 167 (19.5)	266 (69.1) 691 (80.5)	<0.001
**Smoking status**			
Smoker (%) Non-smoker (%)	51 (21.5) 235 (23.4)	186 (78.5) 771 (76.6)	0.545

BMI, body mass index

Microscopic hematuria was seen in 305 (24.54%, 95%CI: 22.14%-26.93%) elderly. 

The prevalence of microscopic hematuria was 27.27% (95%CI: 22.08%-32.47%) in regular users of aspirin and 23.72% (95% CI: 21.02%-26.42%) in non-users of aspirin (P=0.126). 

The prevalence of microscopic hematuria was significantly higher among regular users of aspirin compared to non-users in multivariate analysis (P=0.035, OR=1.40, 95% CI: 1.02-1.92). 

Multiple logistic regression analysis for determining the association of aspirin regular use and presence of microscopic hematuria was shown in table 2. 

The prevalence of microscopic hematuria in the elderly with diabetes was 18.7%, 12.9% in the elderly with fasting blood glucose 180 milligram per deciliter and higher and 21.2% among elderly with FBS less than 180 (P=0.036). 

**Table 2:** Multiple logistic regression analysis of the association of aspirin regular use and presence of microscopic hematuria in elderly (n=1243)

**Variables**	**Microscopic hematuria**		**P-value** *		
	**Yes (n=305)**	**No (n=938)**		**Odds Ratio (95%CI)**	**P-value**
**Aspirin use**					
Yes (%) No (%)	78 (27.3) 227 (23.7)	208 (72.7) 730 (76.3)	0.126	1.40 (1.02-1.92)	0.035
**Gender**					
Male (%) Female (%)	142 (21.1) 163 (28.6)	532 (78.9) 406 (71.4)	0.002	2.25 (1.64-3.08)	<0.001
**Age**					
60-64 65-69 70-74 75-79 80-84 ≥85	113 (24.9) 49 (19.2) 54 (25) 53 (27.7) 24 (28.6) 12 (27.9)	341 (75.1) 206 (80.8) 162 (75) 138 (72.3) 60 (71.4) 31 (72.1)	0.304	1 0.67 (0.46-0.98) 0.91 (0.62-1.35) 1.07 (0.72-1.59) 1.13 (0.66-1.93) 1.09 (0.53-2.27)	0.295 0.040 0.651 0.730 0.660 0.810
**BMI**					
<25 25-29.99 ≥30	126 (30) 118 (22.2) 61 (21)	294 (70) 414 (77.8) 230 (79)	0.006	1 0.66 (0.48-0.89) 0.55 (0.37-0.80)	0.003 0.007 0.002
**DM History **					
Yes (%) No (%)	72 (18.76) 233 (27.2)	313 (81.3) 625 (72.8)	0.001	0.61 (0.45-0.83)	0.002
**Smoking status**					
Smoker (%) Non-smoker (%)	67 (28.3) 238 (23.7)	170 (71.7) 768 (76.3)	0.082	1.81 (1.25-2.64)	0.002

BMI, body mass index. DM, diabetes mellitus.

* Chi-Square test

## Discussion

Our study showed that the prevalence of microscopic hematuria is significantly higher in the elderly who took regular doses of aspirin compared to those who did not use aspirin. The effect of regular use of aspirin on the prevalence of microscopic hematuria was not associated with age, BMI, diabetes and smoking status of participants. Another finding of the present study was the lower rate of hematuria among diabetic participants.

 This was probably due to higher urinary volume in this group of participants that could dilute the urinary specimen. Higher rate of microscopic hematuria in DM patients with lower FBG levels may support this hypothesis. Microscopic hematuria is sometimes difficult to manage (12, 13). While, it could be an early sign of serious urological disorders in a group of patients, no specific disorders are detected at diagnostic investigations in another group (14, 15). In patients with detected cause of microscopic hematuria, urologic malignancies, urolithiasis, infections are the most common etiology. Our study revealed that taking regular doses of aspirin could be a potential cause of microscopic hematuria in the elderly who had no detectable cause for microscopic hematuria at initial work-up. Very few studies have investigated the association between aspirin use and the presence of microscopic hematuria in elderly population. Jeong et al. evaluated the risk of microscopic hematuria in aspirin users among 56632 adults who were screened in a health screening program. Microscopic hematuria was reported in 6.2% of their participants. The authors reported no significant association between low-dose aspirin and microscopic hematuria (6.1% in aspirin users versus 6.2% in non-users, P=0.71) (16). Another study with small population by Culclasure et al. also reported no significant association between aspirin users and microscopic hematuria(17). The first study assessed the adult population, whereas, the population of our study was old people which could be an explanation for the different findings. The latter study has only 69 aspirin-users in its analysis which can cause bias in their findings.

Our study was the first big one to evaluate the association of microscopic hematuria and the regular use of aspirin in elderly population. Based on our findings, regular use of aspirin is accompanied with greater risk of microscopic hematuria in elderly. It is a critical finding since microscopic hematuria may be a sign of benign condition such as urolithiasis or cancer in genitourinary system (18, 19). 

Our study suggests that the clinician should think about aspirin causing microscopic hematuria in the elderly if the workup for microscopic hematuria showed no result. In conclusion, microscopic hematuria in elderly individuals with regular aspirin use is common, furthermore, the clinician should rule out other serious sources.

## Notes

**Funding:** The Vice-Chancellery for Research and Technology of Babol University of Medical Sciences financially supported the study.
